# Editorial:Spotlight on Japan - chemical sciences 2023

**DOI:** 10.3389/fchem.2024.1461284

**Published:** 2024-07-30

**Authors:** Yuji Nishiuchi

**Affiliations:** ^1^ GlyTech, Inc., Kyoto, Japan; ^2^ Graduate School of Science, Tohoku University, Sendai, Japan

**Keywords:** advanced glycation end products (AGEs), carnosine, cysteinyl prolyl ester, glycero-glycophospholipid, post-translational modifications (PTMs), prenylflavonoid, protein synthesis, ubiquitin

An organism is a “mass of molecules and reactions” that work together to form a controlled reaction network. In other words, biological phenomena are chemical reactions involving substances. *Spotlight on Japan - Chemical Sciences 2023* brings together reports on chemical approaches to study substances involved in a wide range of life phenomena from the molecular and cellular level to the ecosystem level. It is hoped that these papers will inform readers and inspire their research.

Advanced glycation end products (AGEs) are substances produced by the non-enzymatic reaction of the reducing ends of sugars with the side-chain amino groups of proteins, Lys and Arg, under oxidative stress, resulting in progressive glycoxidation (Vistoli et al., 2013). They are involved in the onset and progression of various diseases such as diabetes, atherosclerosis and Alzheimer’s disease. Thus, the development of AGE formation inhibitors and AGE degraders may be a promising approach to novel therapeutic drug candidates. Ikeda et al. focused on prenylated flavonoids of the Chinese herbal medicine, *Epimedii Herba* (EH). Icariin, the main active ingredient of EH, has been reported to inhibit neurodegeneration and improve cognitive function in neurological disease. Forty known and 3 novel prenylflavonoid compounds were isolated from EH, and their chemical structures were determined. They proposed the chemical structural features required for the AGE production inhibitory activity of these prenylflavonoids based on their inhibition of the formation of carboxymethylated Lys and Arg, the major antigenic AGE structures. Carnosine (β-Ala-His), which exists in high concentrations in muscle and the brain, also has been shown to exhibit various physiological effects, including antioxidant, antiglycation and fatigue recovery effects. In particular, carnosine is crucial for protection against *in vivo* damage caused by reactive oxygen species. Yoshiya et al. studied the reactivity of carnosine towards singlet oxygen (^1^O_2_) at the molecular level. The end products of ^1^O_2_-mediated carnosine oxygenation were characterized using 2D NMR and other analytical methods, and a cyclic homodimer was identified for the first time as one of the end products of the photooxygenation and without an external nucleophile. Although further research is needed to determine the mechanism by which carnosine inhibits AGE formation and exerts antioxidant effects, their study provides one promising direction. Biological membranes not only physically define the boundaries of cells and, in eukaryotes, the boundaries of each intracellular organelle, they are also selectively permeable boundaries where specific molecules are transported into and out of the cell through embedded proteins, and are closely involved in various biological phenomena. Shimamoto et al. outlined the structure, distribution, function, biosynthesis and chemical synthetic approaches of representative glycero-glycophospholipids. Rare glycolipids, including glycero-glycophospholipids, have recently become increasingly important not only due to their structural interest but also due to their unique biological activities, such as immunostimulatory activity and involvement in translocation of various proteins. Furthermore, by chemically synthesizing a rare glycero-glyco“pyrophospho”lipid, which contains a pyrophosphate instead of a phosphate, they demonstrate that chemical synthesis is a powerful tool for investigating the function of glycolipids because it can provide structurally defined molecules.

Post-translational modifications (PTMs) of proteins increase the functional diversity of the proteome through covalent addition of functional groups or proteins, proteolytic cleavage of regulatory subunits or degradation of entire proteins (Voet et al., 2005). Such PTMs include phosphorylation, glycosylation, ubiquitination, nitrosylation, methylation, acetylation, lipidation and proteolysis, and affect all aspects of cell biology and pathogenesis. Therefore, proper identification and understanding of PTMs is essential in cell biology research and in the treatment and prevention of disease. From this perspective, chemical peptide synthesis, which creates target molecules with a well-defined structure, aids in the elucidation of PTM function at the molecular level. At present the most successful and highly versatile convergent method is native chemical ligation (NCL), which enables chemoselective assembly of unprotected peptides via the generation of a new amide bond. NCL has paved the way for the precise chemical synthesis of large peptides and proteins with diverse PTMs. Ubiquitination is a type of reversible protein modification that regulates protein function and is involved in various biological phenomena such as proteolysis, DNA repair, translation regulation and signal transduction. Izumi et al. reported a highly efficient synthesis of isopeptide-linked ubiquitin peptides using δ-selenolysine-mediated NCL. This method takes advantage of the increased nucleophilicity of a peptide dimer with a diselenide moiety at the δ-position of Lys to achieve a remarkably rapid ligation reaction, and the subsequent deselenization reaction can be performed chemoselectively even in the presence of unprotected Cys. Further applications for the synthesis of various ubiquitinated proteins, such as ubiquitinated glycoproteins and ubiquitinated Cys-containing proteins, are expected. In addition to this, the strategy of ligating recombinant polypeptides with chemically synthesized peptides equipped with PTMs is a powerful approach to synthesize proteins with various PTMs. Okamoto et al. reviewed chemical and enzymatic methods of activating *C*-terminal recombinant polypeptides into peptide-thioesters or thioester-surrogates, which are the key to NCL. Such chemical activation reactions function in a variety of solutions containing organic solvents, chaotropic reagents and/or detergents, while biological methods using intein systems or ligase/carboxypeptidase often have a solubility problem due to the aggregability of polypeptides and cannot adapt to solution conditions that would improve it. Chemical methodologies can be categorized as 1) Cys side chain modification and 2) activation via N-S acyl shift. They developed *C*-terminal Xaa-Cys-NHR (R = amino acid or peptide) and Xaa-Cys-Gly-Cys sequences applicable to each of the two pathways and the conditions for converting them to Xaa-thioesters and synthesized glycoproteins interleukin-13 and inducible T-cell costimulatory (CD278), respectively. NCL is a useful method not only for protein synthesis but also for library construction, especially for cyclic peptides. To construct a cyclic peptide library of the RGD integrin binding motif as a model, Hojo et al. applied their previously developed cysteinyl prolyl ester method to the cyclization of Cys-RDG-peptide-Cys-Pro-esters, in which the two Cys and Pro in the CPC moiety have different configurations. Regardless of configuration differences, cyclization proceeded efficiently to yield cyclic peptides that were then subjected to Cys desulfurization or alkylation, resulting in a large diversity of analogs with different conformations of the CPC moiety. This approach is expected to be useful in the search for protein-protein interaction inhibitors.
